# Supplementary material for: Intravital imaging of pulmonary lymphatics in inflammation and metastatic cancer
Source: bioRxiv. 2024 Sep 17:2024.09.12.612619. Preprint. [Version 1] doi: 10.1101/2024.09.12.612619 (PMC11430110; doi:10.1101/2024.09.12.612619)
Supplement: Supplement 9 [file NIHPP2024.09.12.612619v1-supplement-9.pdf]

bioRxiv preprint doi: https://doi.org/10.1101/2024.09.12.612619; this version posted September 17, 2024. The copyright holder for this preprint (which was not certified by peer review) is the author/funder, who has granted bioRxiv a license to display the preprint in perpetuity. It is made available under aCC-BY 4.0 International license.

Supplemental material

Video 1 Effects of changing ventilator settings on pulmonary lymphatic valve function and afferent lung lymph flow

Video 2 Effect of changing ventilator tidal volume on lymph flow within pulmonary lymphatics following LPS-induced acute lung inflammation

Video 3 Leukocyte dynamics and diversity within pulmonary lymphatics after LPS-induced acute lung inflammation

Video 4 Effect of pertussis toxin on leukocyte flow within pulmonary lymphatics following LPS-induced acute lung inflammation

Video 5 Effect of knockout of *Ccr7* on leukocyte flow within pulmonary lymphatics following LPS-induced acute lung inflammation

Video 6 Effect of Ccr7 blocking antibody treatment on leukocyte flow within pulmonary lymphatics following LPS-induced acute lung inflammation

Video 7 Pulmonary lymphatic trafficking of leukocytes, cancer cell material and cancer cells following lung metastasis of B16.F10 melanoma cells

Supplementary Data File 1 3D model of thoracic window

bioRxiv preprint doi: https://doi.org/10.1101/2024.09.12.612619; this version posted September 17, 2024. The copyright holder for this preprint (which was not certified by peer review) is the author/funder, who has granted bioRxiv a license to display the preprint in perpetuity. It is made available under aCC-BY 4.0 International license.

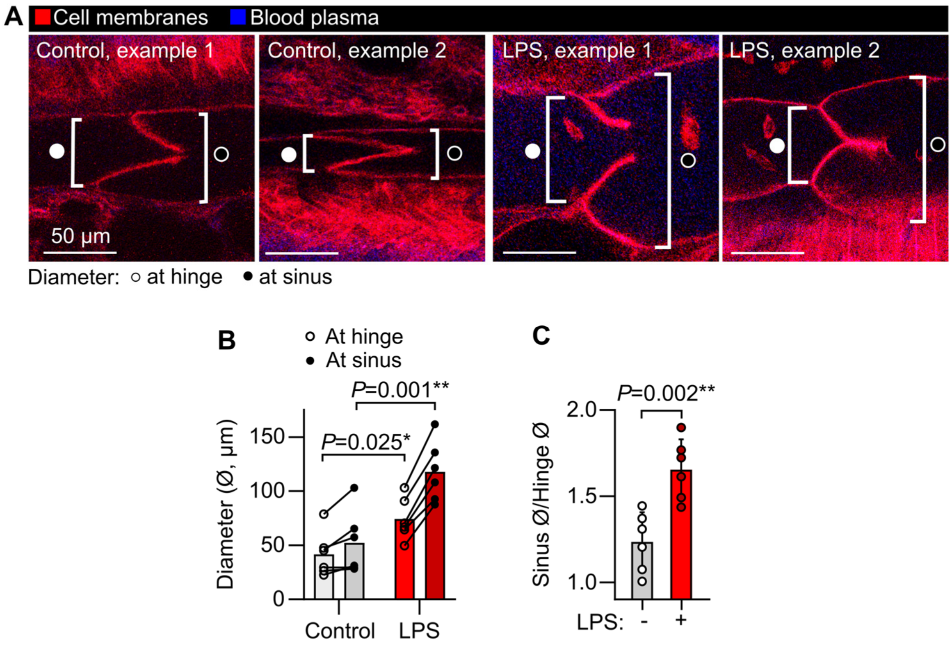

Figure S1: Measurement of pulmonary lymphatic distension in LPS-induced acute lung inflammation. (**A**) Representative images of pulmonary lymphatic valves from steady state controls and LPS-treated *Rosa26*^{mTmG} mice showing approach for measuring lymphatic diameter. (**B**) Lymphatic vessel diameters at valve hinges and at sinuses immediately downstream of valves. (**C**) Sinus diameters divided by hinge diameters showing relative distension of sinuses. Graphs show means ± SEM. *P*-values are from: (**B**) repeated measures, 2-way ANOVA with Holm-Šídák test for effect of LPS within vessel region groups; or (**C**) unpaired, 2-tailed t-test. Group sizes: n=6.

bioRxiv preprint doi: https://doi.org/10.1101/2024.09.12.612619; this version posted September 17, 2024. The copyright holder for this preprint (which was not certified by peer review) is the author/funder, who has granted bioRxiv a license to display the preprint in perpetuity. It is made available under aCC-BY 4.0 International license.

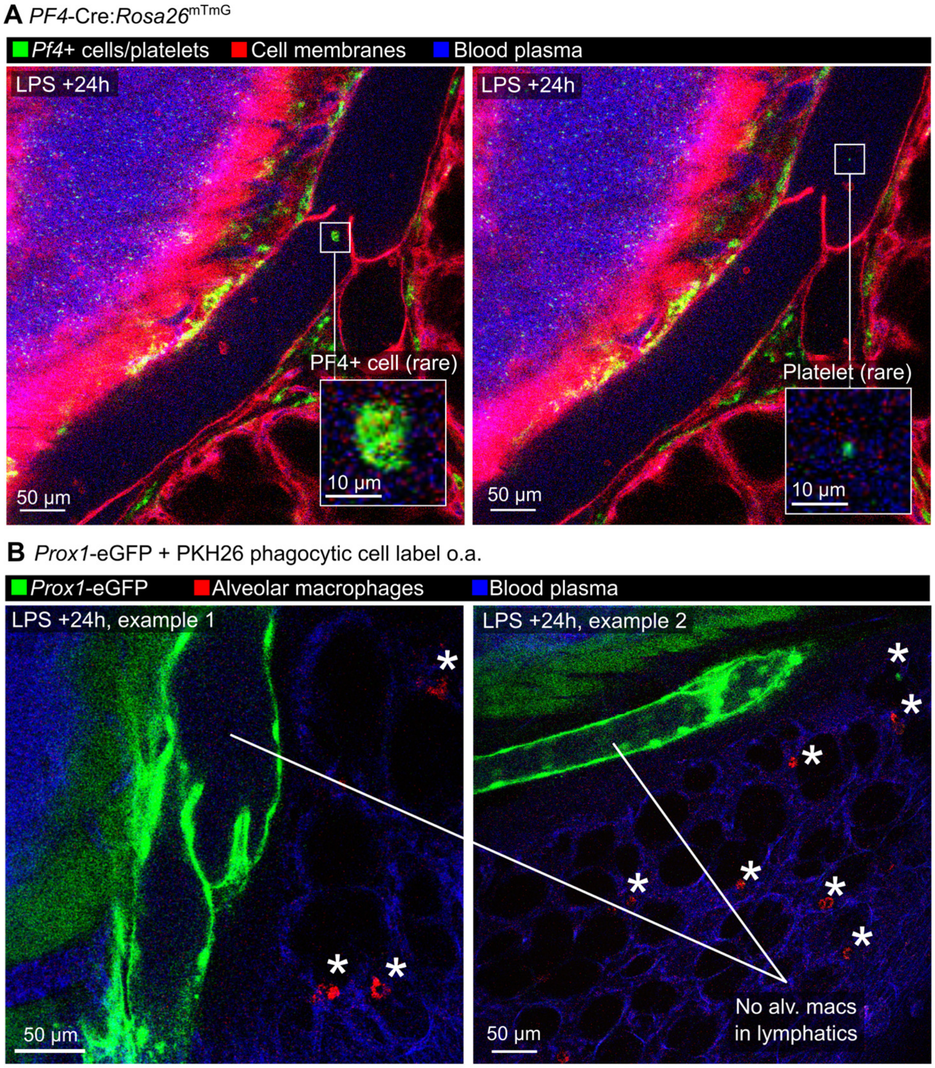

Figure S2: Imaging of pulmonary lymphatics in LPS-treated *Pf4*-Cre:*Rosa26*^{mTmG} mice and *Prox1*-eGFP mice given PKH26-PCL to label alveolar macrophages. (**A**) Intravital images of an LPS-treated *Pf4*-Cre:*Rosa26*^{mTmG} mouse showing platelets in blood vessels and recombined cells in bronchovascular cuff spaces but only very rare recombined cells and possible platelets in lymph. (**B**) *Prox1*-eGFP mice were given an o.a. dose of PKH26-PCL dye to label alveolar macrophages, then 5 days later mice were given o.a. LPS. Intravital imaging at 24 hours after LPS treatment showed labeling of alveolar macrophages (alv macs, asterisks) in alveoli but not in lymphatic vessels.

bioRxiv preprint doi: https://doi.org/10.1101/2024.09.12.612619; this version posted September 17, 2024. The copyright holder for this preprint (which was not certified by peer review) is the author/funder, who has granted bioRxiv a license to display the preprint in perpetuity. It is made available under aCC-BY 4.0 International license.

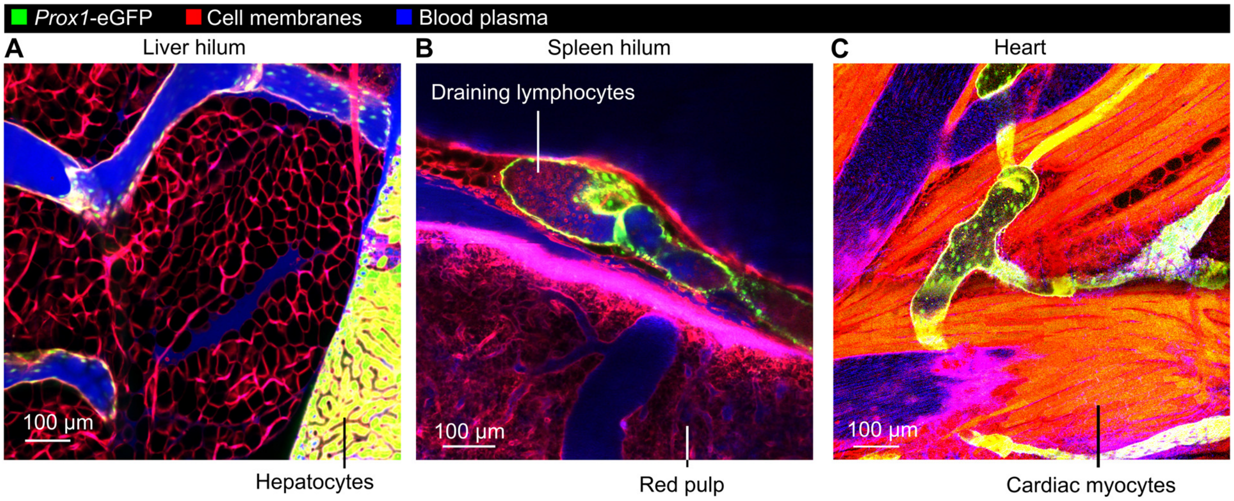

Figure S3: Stabilized imaging of lymphatic vessels draining the liver, spleen and heart. *Prox1*-eGFP:*Rosa26*^{mTmG} mice were given Evans blue i.v. prior to stabilized intravital imaging of: (**A**) the hilum of the liver; (**B**) the hilum of the spleen; and (**C**) the ventricular wall of the heart. Note free movement of Evans blue-labeled plasma proteins into liver and spleen draining lymphatics, likely due to the fenestrated endothelium lining blood vessels in these organs, as well as many leukocytes with lymphocyte morphology draining from the spleen, a secondary lymphoid organ.
